# Therapeutic Landscape of FOXM1 in Triple-Negative Breast Cancer and Aggressive Solid Cancers

**DOI:** 10.3390/cancers16223823

**Published:** 2024-11-14

**Authors:** Sayra Dilmac, Zuhal Hamurcu, Bulent Ozpolat

**Affiliations:** 1 Department of Nanomedicine, Houston Methodist Research Institute, Houston, TX 77030, USA; sdilmac@houstonmethodist.org; 2 Department of Medical Biology, Faculty of Medicine, Erciyes University, Kayseri 38030, Turkey; zhamurcu@erciyes.edu.tr

**Keywords:** FOXM1, cell cycle, triple-negative breast cancer, therapy, brain tumors, lung cancer, breast cancer, inhibitor, siRNA, microRNA

## Abstract

Triple-negative breast cancer (TNBC) is one of the most aggressive forms of breast cancer, lacking common treatment targets such as estrogen (ER), progesterone (PR), and HER2 receptors. This subtype is associated with significant heterogeneity, chemoresistance, early recurrence, metastasis, and poor patient survival. FOXM1 is a cancer-promoting transcription factor that plays a critical role in TNBC and other highly aggressive cancers by driving cell proliferation, invasion, metastasis, and drug resistance. In TNBC, mutations in the TP53 gene—detected in approximately 80% of patients—lead to the overexpression of FOXM1, making it a promising therapeutic target. Beyond TNBC, FOXM1 is implicated in other solid cancers, such as brain (glioblastoma), lung, and pancreatic cancers, and is considered an Achilles’ heel of aggressive cancers. Despite its potential as a therapeutic target, there are currently no FDA-approved FOXM1 inhibitors, and none have advanced to clinical trials. This review explores the role of FOXM1 in cancer progression and highlights the current status of efforts to develop effective FOXM1 inhibitors.

## Simple Summary

FOXM1 is a pro-oncogenic transcription factor that is often upregulated in triple-negative breast cancer and aggressive solid cancers. FOXM1 drives cell proliferation, invasion, metastasis, drug resistance, and tumor growth and progression, and its expression is associated with poor prognosis and shorter patient survival. Therefore, FOXM1 is considered as an emerging therapeutic target. This review highlights the role of FOXM1, the mechanisms regulating its expression, its potential as a novel molecular target in TNBC and solid tumors, and attempts to develop FOXM1 inhibitors.

## 1. Introduction

Forkhead box (FOX) proteins are a family of proteins that have a conserved FOX domain and extra FOX protein–protein interaction (PPI) domains or regions that play an active role in many cellular processes, including cell growth, differentiation, and embryogenesis [1]. Forkhead family members play essential roles in many biological processes, whereby the most notable are embryogenesis, apoptosis, differentiation, longevity, transformation, proliferation, tumorigenesis, and homeostasis. The mammalian transcription factor, Forkhead box M1 (FOXM1), a member of the Forkhead transcription factor family, is strictly evolutionary conserved and contains a 100 amino acid-long DNA-binding domain called the Forkhead or winged-helix domain [2]. The *FOXM1* gene is localized on the p12.13.33 chromosome in humans [1,3]. FOXM1 is highly abundant in adult organs with high proliferation potential, such as the small intestine, colon, testes, and thymus. Lower levels of FOXM1 expression are found in the lungs, spleen, and ovaries, where cells with less propensity to divide are present [4].

Recent studies indicated that FOXM1 functions include regulation of cell proliferation, migration, angiogenesis, vascular permeability, oxidative stress, and inflammation [3,5,6]. In addition, FOXM1 is involved in the regulation of the cell cycle phases, including G1-S, G2, and M phase transitions, mitosis, and cytokinesis [7]. In normal physiology, FOXM1 plays an essential role in maintenance of regenerative cell proliferation, particularly during adult tissue repair [8]. The increased FOXM1 expression is an important driver of cell proliferation, autophagy, migration, invasion, tumor growth, and progression, regulating the expression of targeted genes such as cyclin B1/D1, STAT3, Aurora A/B, BRCA2, Beclin1, PLK, RAD51, MMP2, VEGF-A, and integrin B. *FOXM1* is amplified in 5.6% of breast cancers (BCs), 42% of non-Hodgkin lymphomas, and 58% of malignant peripheral nerve sheath tumors. Amplification of the FOXM1 gene is increased in TNBC (33.3%) [9]/basal-type BCs and three subtypes of non-Hodgkin lymphoma [1]. Additionally, FOXM1 is expressed in all stages of breast cancer and all breast cancer subtypes, including TNBC, ER+, and HER2+ [10]. In the following sections, the role of *FOXM1* in cellular processes and tumorigenesis is discussed in detail.

## 2. Molecular Mechanisms Regulating FOXM1 Expression and Activity

### 2.1. Genetic and Transcriptional Regulation of FOXM1 Expression

The p53 tumor suppressor protein is a major regulator/suppressor of FOXM1 activity. As a transcription factor, p53 controls cellular responses to stress by modulating processes like cell cycle progression, DNA repair, senescence, and apoptosis. TP53 mutations occur in 18–25% of primary breast cancers, approximately 84% of TNBC, and 50% of all human cancers. These mutations prevent apoptosis and drive malignant transformation [11]. Tumors with TP53 mutations are highly invasive, poorly differentiated, and resistant to chemotherapy. Most importantly, p53 negatively regulates FOXM1 expression through transcriptional repression. FOXM1 expression is upregulated by partial deletion or inactivation of p53 [11]. When DNA damage occurs due to genotoxic drug treatment, ATM and p53 suppress FOXM1 mRNA expression via the E2F1 transcription factor, which binds to a FOXM1 promoter and drives its expression [12]. However, in the absence of functional p53, FOXM1 expression is induced through the ATM and E2F1 pathways [13,14]. Additionally, p21, which is regulated by p53, inhibits FOXM1 through the p53/Rb/E2F interaction [13]. Nutlin-3, which activates p53, has been shown to degrade FOXM1 mRNA, further highlighting p53’s role in FOXM1 suppression. Mutant p53 can induce FOXM1 expression in certain cancers, including endometrial and ovarian cancer cell lines [15]. In TNBC, MELK (maternal embryonic leucine zipper kinase) is highly expressed and regulated by wild-type p53. WT p53 suppresses MELK expression by inhibiting FOXM1 binding to the MELK promoter [16].

The retinoblastoma (Rb) tumor suppressor also regulates FOXM1. When Rb forms a complex with FOXM1, it represses GATA3 and PTEN, leading to the accumulation of poorly differentiated, cancer stem-like, and pro-metastatic cells. Disrupting this FOXM1/Rb interaction alters the tumor microenvironment, reducing metastatic potential [17].

**Figure 1:**
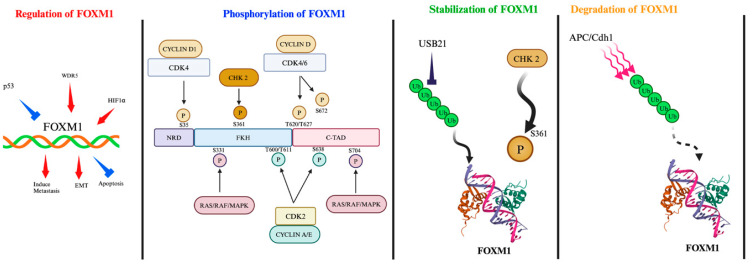
Mechanisms regulating FOXM1 activity and its downstream signaling pathways. Several genes and transcription factors such as p53, WDR5, and HIF-1 α regulate FOXM1 expression, leading to increased cell invasion and metastasis through EMT and inhibition of apoptosis, and promote cell survival. The activity of FOXM1 is regulated by phosphorylation at various residues by CDK/cyclin, RAS/RAF/MAPK, and CHK2 kinases and proteasomal degradation through USB21, which stabilizes FOXM1 via inhibition of the ubiquitylation process while APC ubiquitylates and induces FOXM1 degradation.

Several other transcription factors and signaling pathways regulate FOXM1 expression. GLI1, downstream of the Hedgehog pathway, along with CTCF, CREB, STAT3, and E2F, bind to the *FOXM1* promoter to enhance its expression (Figure 1) [17]. Furthermore, Twist1 and p300 have been shown to regulate *FOXM1* mRNA expression [18], while HIF-1α-induced FOXM1 expression has been linked to stress adaptation via the ubiquitination by the VHL E3 ligase complex [19]. HIF-1α-induced FOXM1 expression (Figure 1) has been linked to increased resistance to apoptosis in cancers like hepatoma, contributing to tumor progression [20].

### 2.2. Post-Transcriptional Regulation of FOXM1 Protein Expression

Phosphorylation plays a key role in regulating FOXM1’s transcriptional activity throughout the cell cycle (Figure 1) [21], with various phosphorylation dynamics [22]. FOXM1 is inactivated by hypo-phosphorylation in the G1/S phase, and its phosphorylation increases from the S phase to the G2/M transition. In the late G1 phase, cyclin D–CDK4/6 (Cyclin-Dependent Kinase 4/6) complexes phosphorylate FOXM1 on T620, T627, and S672 residue sites, triggering the G1 to S cell cycle transition [23]. In the late S and G2/M phases, phosphorylation of FOXM1 on both S331 and S704 via Raf/MEK/MAPK stimulates the nuclear translocation of FOXM1, thereby enabling its transcriptional activity [21]. Checkpoint kinase 2 (Chk2) phosphorylates FOXM1 at S361, inhibits its degradation, and increases the transcription of the *XRCC1* (X-ray repair cross-complementing protein 1) and *BRCA2* genes required for DNA damage repair [24]. Finally, during the G2 phase of the cell cycle, cyclin A/E–CDK2 complexes phosphorylate FOXM1 at the T600, S638, and specifically T611 sites, which reduces TAD (transactivation domain) suppression by NRD (N-terminal repressor domain) and restores TAD transactivation activity [25,26]. Furthermore, recent in vitro studies have shown that several tumor suppressor microRNA molecules (i.e., miR34a, miR-802, and miR-877-5p) have binding sites at 3′-UTR of mRNA FOXM1 and inhibit its expression and translation in in vitro and in vivo studies. Some of these miRNAs, such as miR34a and miR-877-5p, are reduced or lost in various cancers such as breast cancer, liver cancer [27], cervical cancer [28], stomach cancer [29], and lung cancer [30], potentially leading to overexpression of FOXM1.

### 2.3. FOXM1 Degradation by Stability Regulation

Targeting FOXM1 degradation is an emerging therapeutic strategy. Ubiquitin-specific protease 21 (USP21) is a deubiquitinating enzyme (DUB) that stabilizes FOXM1 by removing ubiquitin, protecting it from proteasomal degradation (Figure 1). Inhibiting USP21 could therefore represent a novel approach to reducing FOXM1 levels [31].

Moreover, FOXM1 is regulated by post-translational modifications like SUMOylation, which enhances APC/Cdh1-dependent ubiquitin-mediated degradation, reducing FOXM1 activity. Small Ubiquitin-like Modifier (SUMO) proteins are small proteins that covalently bind to other proteins and modify their functions. SUMOylation is a crucial post-translational modification (PTM) that regulates the activity, stability, and other PTMs of FOXM1 [17]. SUMOylation of FOXM1 can enhance APC/Cdh1-dependent ubiquitin-mediated degradation (Figure 1), leading to reduced transcriptional activity of FOXM1c [32]. Long noncoding RNAs (lncRNAs) such as PVT1 also play a role in stabilizing FOXM1 by binding to the protein and preventing its degradation. Transcriptional activation of FOXM1 occurs by the phosphorylation of FOXM1 at T596 following the binding of CDK–cyclin complexes [33]. Elucidating the interactions between FOXM1 and other proteins will facilitate the design of short peptides and small molecules for targeting FOXM1.

DNA damage signaling pathways further regulate FOXM1 protein stability [24]. FOXM1 is phosphorylated at S361 and stabilized by DNA damage-induced Chk2. This increased stability of the FOXM1 protein in response to DNA damage [24] leads to expression of the DNA repair genes such as XRCC1 and BRCA2.

In summary, FOXM1 regulation is multifaceted, involving a complex network of genetic, transcriptional, post-transcriptional, and post-translational mechanisms. Understanding these pathways provides valuable insights into potential therapeutic strategies for targeting FOXM1 in cancer treatment.

## 3. Role of FOXM1 in Cellular Processes

### 3.1. Role of FOXM1 in the Cell Cycle

**Figure 2:**
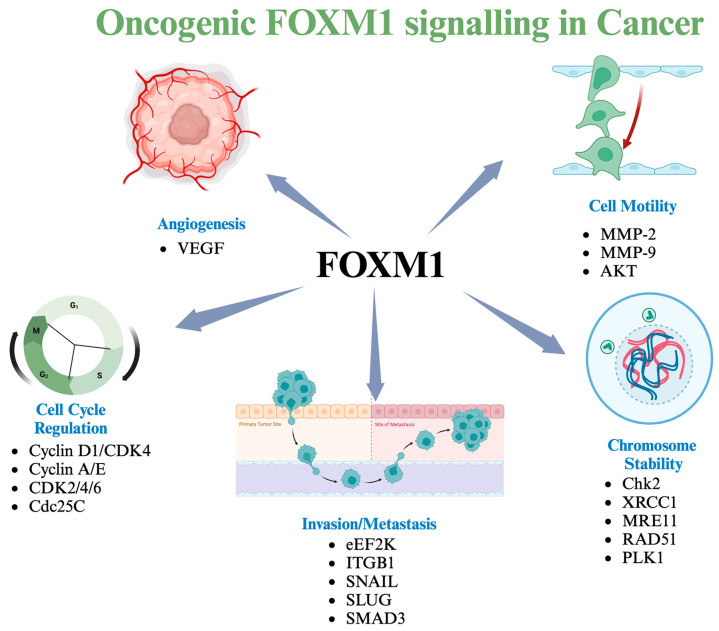
The FOXM1 signaling axis provides a major oncogenic drive in TNBC and other solid cancers. FOXM1 promotes the expression of genes that drive cell cycle progression, migration, invasion, metastasis, and angiogenesis and alters chromosomal stability, all of which contribute to tumor growth, metastasis, and progression.

FOXM1 activity is tightly controlled to ensure expression of downstream effectors and prevent uncontrolled cell proliferation (Figure 2). It is minimally expressed in non-dividing cells, but becomes highly active during the late G1 phase and continues to increase through G2/M phases. FOXM1 activity is directly linked to its phosphorylation status, with phosphorylation being essential for its stabilization and activation [2,5]. As cells transition from G2 to M, FOXM1 expression peaks, and its phosphorylation ensures sustained activity throughout the cell cycle. Conversely, FOXM1 is dephosphorylated as cells exit mitosis, marking the end of its activity [22].

The first phosphorylation of FOXM1 (S251) occurs in G1, and it is then sequentially phosphorylated by multiple protein kinases, including CDK1 and PLK, CDK–cyclin complexes and mitogenic kinases, during the S and G2/M phases of the cell cycle. S215 phosphorylation is required for nuclear localization of FOXM1 [22]. The cyclin D1/CDK4 complex phosphorylates FOXM1 at S35 preceding the full activation of the FOXM1 transcriptional function by the following phosphorylation cascade [23,34]. During the G2 phase, the cyclin A/E–CDK2/4/6 complex phosphorylates FOXM1 at T600, T611, and S638, enabling complete FOXM1 transactivation activity. These successive phosphorylation events reduce the suppression of TAD by NRD and ensure the continuation of FOXM1 activity [10]. In the G2/M phase, FOXM1 becomes fully hyperphosphorylated (T600, T611, and S638), as necessary for its activation. FOXM1 enables transcription of G2/M-specific genes, such as cyclin B, Cdc25B, Aurora B, PLK1, survivin, CENP-A, CENP-B, and CENP-F, which are key regulators of mitosis and chromosomal segregation [2,22,23]. FOXM1 maintains mitosis progression and chromosomal stability during the G1/S and G2/M transitions. When FOXM1 is downregulated, mitosis does not progress beyond prophase, indicating that FOXM1 is required for mitosis progression. Furthermore, loss of FOXM1 causes a delay in G2/M progression, mistakes in chromosome segregation, and cell cycle abnormalities, including failure of cytokinesis [35,36,37].

The role of FOXM1 in the early stages of tumorigenesis became clearer with the observation that hepatic *FOXM1b* expression in transgenic mice promotes cell proliferation in pre-neoplastic and early neoplastic lesions, and has a less clear effect on hepatocellular carcinoma development [38], as FOXM1 deficiency increases nuclear p27/Kip1 in normal hepatocytes. In addition, the decreased level of Cdc25C indicates that FOXM1 cooperates with other cell cycle regulators and oncogenes to induce proliferation in cancer cells [39]. These findings further suggest that FOXM1 plays a critical role in cell cycle progression. Thus, defects in FOXM1 regulation confer proliferative advantages to cells but render them more susceptible to tumor-assisted transformation by oncogenes. Recent data also suggest that FOXM1 has a positive autoregulatory capability; FOXM1 regulates its own transcription and can bind to its own promoter [40].

FOXM1 expression is inhibited by proteasome inhibitors [41]. Considering that the main task of proteasome inhibitors is to stabilize the expression of cellular proteins, there is a possibility for proteasome inhibitors to be used as negative regulators of FOXM1 (NRFMs) [42]. In the absence of proteasome inhibitors, the transcriptional activity of FOXM1 is controlled by a positive feedback loop. Following proteasomal inhibition, the NRFM process becomes active and directly or indirectly inhibits FOXM1 transcriptional activity, resulting in inhibition of its positive autoregulation or direct suppression of FOXM1 expression [40,42].

### 3.2. FOXM1 and Angiogenesis

Angiogenesis, the formation of new blood vessels, is crucial for tumor growth and progression, supplying oxygen and nutrients. FOXM1 binds to the Forkhead elements (FHRE) of the VEGF promoter, enhancing VEGF expression (Figure 2). A positive correlation between FOXM1 and VEGF expression has been observed in various cancers, including breast, brain, and stomach cancers. Suppression of FOXM1 expression in pancreatic cancer cells suppresses VEGF secretion [43,44]. Knockdown of FOXM1 expression by siRNA in glioblastoma cells inhibits VEGF secretion and capillary tube formation. Similar findings in gastric carcinomas and hepatocellular carcinoma (HCC) further support that FOXM1 is required for VEGF-induced angiogenesis in solid tumors [43,44].

### 3.3. FOXM1 and Cell Motility

The ability of tumor cells to leave their environment and move toward the surrounding tissues, migrate, and metastasize is a distinguishing feature of aggressive tumors. During cancer cell migration, various components of the basement membrane and extracellular matrix (ECM), the main support elements of the cell, are facilitated by multiple enzymes (such as proteases) and matrix metalloproteinases (MMPs) [45]. Among these proteases, MMP-2 and MMP-9 are frequently associated with tumor cell migration, invasion, and metastasis. Inhibition of FOXM1 has been shown to reduce the expression of MMP-2 and MMP-9, and suppress the migration and invasion of tumor cells (Figure 2) [39]. Similarly, FOXM1 regulates cell migration and invasion via MMP-2 and MMP-9 in tumors such as glioblastoma, colorectal carcinoma, and breast carcinoma [46,47]. There is a Forkhead consensus region in the MMP-2 promoter, suggesting that FOXM1 can directly regulate MMP-2 expression at the transcriptional level [47]. Furthermore, FOXM1 indirectly regulates MMP-9 expression through its downstream target JNK [48]. In the absence of the tumor suppressor Arf, overexpression of FOXM1 directly supports metastatic transformation by inducing EMT via Akt. In addition, FOXM1 impairs cytoskeleton stability by stathmin modulation, which is responsible for microtubule dynamics [49]. FOXM1 promotes epithelial-to-mesenchymal transition (EMT), a key process of the metastatic process, whereby the WNT/ β-catenin signaling pathway plays a critical role [50].

### 3.4. FOXM1 and Immune Escape

Checkpoint inhibitors targeting PD-L1, PD1, and CTLA4 have been shown to enhance the immune response in solid tumors and revolutionize cancer immunotherapy [51,52]. PD-L1, PD1, and CTLA4 suppress T-cell function and help cancer cells escape immunosurveillance. It has been shown that FOXM1 selectively increases PD-L1 expression by directly binding to the PD-L1 promoter [53]. In vitro and in vivo inhibition of FOXM1 reduced PD-L1 expression, indicating that targeting FOXM1 is a promising strategy for inhibiting PD-L1 expression [53].

## 4. FOXM1 and Solid Tumors

### 4.1. Triple-Negative Breast Cancer

TNBC represents ~15–20% of all breast cancers (50,000 cases/year in the US). TNBC has the highest death rate (5-year survival 8–16%) of all breast cancer subtypes and is characterized by a lack of common therapeutic targets like estrogen receptor (ER^−^), progesterone receptor (PR^−^), and human epidermal growth factor receptor 2 (HER2^−^). These tumors are characterized by early metastasis, drug resistance, and high rates of relapse due to a lack of effective therapeutic options [54,55]. TNBC patients with metastatic disease have a life expectancy of only about 13 months, a 5-year death rate of ~40% with regional cancer (stage 2), and 90% of them present with distant metastasis (stage 4) [56]. Chemotherapy, surgery, and radiation therapy remain the only treatments for TNBC, providing major motivation for the development of effective therapeutics to improve patient survival. Currently, there are no effective therapeutics that target oncogenic pathways in TNBC. In April 2020, the U.S. Food and Drug Administration (FDA) approved the first targeted therapy in metastatic TNBC, sacituzumab (govitecan), a Trop-2-receptor directed antibody conjugated with a chemotherapeutic (a topoisomerase inhibitor). However, the median duration of response was 7.7 months, and the majority of patients (83%) did not maintain a response longer than 12 months [55], indicating the need for novel, more effective therapeutic strategies for TNBC patients.

Recently, a landmark study involving comprehensive molecular analysis of 500 human breast tumors revealed the *FOXM1* gene signature as one of the most critical molecular drivers of TNBC [57]. FOXM1 expression is associated with an advanced tumor stage, high proliferation rate, and poor prognosis, suggesting that FOXM1 is a new prognostic marker for patients with breast cancer [5,58]. In addition, compared to normal breast tissue and tumor tissues, FOXM1 is associated with ER expression in breast cancer, resistance to hormone therapy, and poor prognosis [59]. *FOXM1* expression is also associated with a larger tumor size, lymphovascular invasion, lymph node metastasis, and a higher stage in ER+ hormone therapy-resistant breast cancer [60]. Recent studies have also shown that FOXM1 dysregulation is associated with cancer progression and the development of drug resistance in breast cancer [61].

FOXM1 is highly overexpressed in TNBC cells compared to normal breast cells and induces cell proliferation, colony formation, migration, and invasion [62]. FOXM1 expression is associated with poor prognosis in patients with TNBC [63]. Through our own work, we were able to demonstrate that in vivo inhibition of FOXM1 by siRNA FOXM1 injections can suppress tumor growth in TNBC tumor xenograft models in mice [62], suggesting that FOXM1 is a clinically significant molecular target driving the TNBC tumorigenesis. FOXM1 overexpression in TNBC confers an apoptosis-resistant phenotype associated with overexpression of X-linked inhibitor of apoptosis protein (XIAP) and survivin (an anti-apoptotic protein) [64]. Importantly, inhibition of FOXM1 expression sensitizes cells to chemotherapy (doxorubicin) [65].

In TNBC cells, FOXM1 binds to the promoters of *eEF2K* (eukaryotic elongation factor 2 kinase) and *ITGB1* (integrin subunit beta 1) genes and transcriptionally drives their expression and cell cycle, migration/invasion, and survival through cyclin D1, Src, and the MAPK/ERK signaling pathways [62,66]. In addition, FOXM1 promotes EMT in breast cancer by directly binding the SLUG promoter and driving its expression [67]. FOXM1 interacts with SMAD3 and induces breast cancer metastasis by supporting the activation of TGF-*β* [68]. FOXM1 also binds to the promoters of autophagy-related Beclin-1 and LC3 and regulates their expression [69]. Beclin-1 and LC3 are overexpressed in breast cancers and are associated with poor survival in TNBC patients, suggesting that targeting FOXM1 may suppress the tumor-promoting autophagic process [69].

Subsequent studies demonstrated that FOXM1 mediates serotonin (5-hydroxytryptamine)/serotonin receptor signaling through serotonin receptor 5-HT7, which induces the proliferation and migration of TNBC cells. Clinically, its expression is associated with a poor prognosis and shorter patient survival in TNBC patients. Inhibition of the 5-HT7 receptor reduces FOXM1 expression and cell proliferation, suggesting that targeting 5-HT7 receptor/FOXM1 signaling may be a new therapeutic strategy for treating TNBC [70].

**Figure 3:**
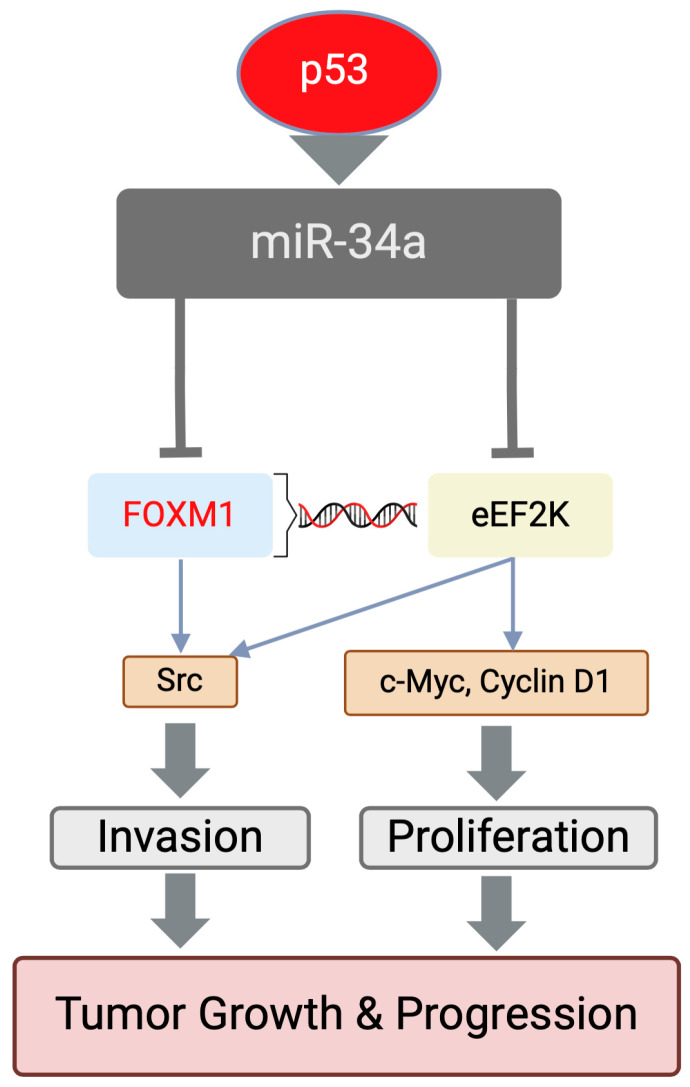
miR-34a suppresses the FOXM1/eEF2K oncogenic axis by specifically binding to their mRNA at 3′-UTR, leading to inhibition of cell proliferation and invasion.

Through our own work, we found that expression of miR-34a, a p53-induced miRNA, is reduced or lost in TNBC patient tumors and cell lines and that reduced miR-34a expression is associated with shorter patient survival. We demonstrated that miR-34a acts as a tumor suppressor in TNBC by suppressing the FOXM1/eEF2K oncogenic axis (Figure 3). Expression of miR-34a in in vitro models of TNBC cells suppressed cell proliferation and invasion by specifically binding and targeting 3′-UTR of FOXM1/eEF2K mRNAs. Notably, miR-34a expression recapitulated the effects of inhibition of *eEF2K* and *FOXM1*, the transcription factors for *eEF2K,* and overexpression of *eEF2K* and *FOXM1* rescued the effects of miR-34a, suggesting that *FOXM1/eEF2K* are the major downstream targets mediating the tumor-suppressive effects of miR-34a.

### 4.2. ER^+^ and HER^+^ Breast Cancer

FOXM1 has been implicated in estrogen signaling in ER^+^ BC cells, in which FOXM1 is a transcriptional target of ERα and plays a critical role in the sensitivity and resistance to endocrine (anti-estrogen) therapy in breast cancer [71]. FOXM1 is overexpressed in tamoxifen-resistant ER^+^ BC cells, and its inhibition sensitizes cells to tamoxifen. Inhibition of FOXM1 in MCF-7 cells almost completely inhibits ERα expression, suggesting a reciprocal activation relationship between the two molecules [72]. In contrast, ectopic ERβ1 expression decreases FOXM1 protein and mRNA expression in ERα-positive cancer cells, suggesting that ERβ1 represses ERα-dependent FOXM1 transcription [73]. Such a relationship was not seen in ER^-^ cells. In cisplatin-resistant MCF-7-CISR cells compared to their parental MCF counterparts, FOXM1 expression (both protein and mRNA) is elevated [74]. Inhibition of FOXM1 following siRNA treatment reverses cisplatin resistance in MCF-7-CISR breast cancer cells, suggesting that FOXM1 is a potential therapeutic target in cisplatin-resistant ER^+^ breast cancer [74].

In HER2^+^ breast cancers, FOXM1 has emerged as a key diagnostic marker [75] and a critical downstream target of HER2 signaling. HER2 regulates FOXM1 at both the transcriptional (mRNA) and translational (protein) levels, and FOXM1 plays an essential role in HER2-mediated signaling and patient prognosis. Importantly, FOXM1 is a potential therapeutic target, particularly in HER2^+^ breast cancers that are resistant to HER2-targeted therapies [75].

### 4.3. Brain Tumors

Glioblastoma multiforme (GBM) is a highly aggressive brain tumor with poor survival outcomes, often due to resistance to chemotherapy and radiotherapy [76]. High FOXM1 expression is associated with a reduced responsiveness to these treatments [76]. FOXM1 induces Sox2, a key stem cell regulator, in GBM cells, and both proteins are upregulated after radiotherapy [76]. Inhibition of FOXM1 sensitizes GBM cells to radiotherapy by downregulating genes involved in DNA repair, such as MRE11, RAD51, and PLK1, which are critical for chromosomal instability-induced radiotherapy resistance [77]. In addition to its role in radiotherapy resistance, FOXM1 contributes to chemotherapy resistance in GBM through its transactivation of Replication Factor C Subunit 5 (RFC5), a DNA repair protein. Inhibition of FOXM1 or RFC5 sensitizes glioma cells to Temozolomide (TMZ), a first-line standard therapeutic drug, indicating that FOXM1 induces TMZ resistance. On the other hand, the combination of TMZ with a FOXM1 inhibitor, thiostrepton, has been shown to enhance TMZ-induced apoptosis in glioma cells [78]. In addition to its role in chemotherapy and radiotherapy resistance, FOXM1 is involved in reducing autophagic death in glioma cells by regulating the expression of Ubiquitin-conjugating enzyme E2C (UBE2C), which plays a critical role in the development of gliomas. FOXM1 increases UBE2C expression, and a high FOXM1/UBE2C ratio correlates with poor prognosis in GBM patients. In glioma cell lines (i.e., U87-MG, U251, ln18, and U373), UBE2C, a known glioma tumor progression protein, harbors two FOXM1 binding sites on its promoter, strongly suggesting that it could be another direct transcriptional target of FOXM1. Because inhibition of UBE2C promotes autophagic cell death in glioma cells, the FOXM1/UBE2C axis could be essential in developing resistance to therapies, and its targeting holds therapeutic value [79]. FOXM1 has been shown to increase the nuclear localization of the transcription factor GLI, a key player in GBM progression [80], by upregulating Importin-7 (IPO7), which facilitates GLI1 nuclear import. Furthermore, recent studies have indicated the existence of a positive feedback regulatory loop between FOXM1 and GLI1 [80]. Additionally, FOXM1 activates ADAM17 expression, which triggers the EGFR/AKT signaling pathway, contributing to EMT phenotype development and GBM progression. These findings suggest that targeting the FOXM1/GLI1 and FOXM1/ADAM17 axes are promising therapeutic strategies for GBM [81].

### 4.4. Lung Cancer

Lung cancer, particularly non-small cell lung cancer (NSCLC), is the leading cause of cancer-related deaths, with an estimated overall survival (OS) rate of 15 months [82]. Overexpression of FOXM1 in patients with NSCLC is associated with poor prognosis, metastasis, and chemoresistance [83]. In mouse models, FOXM1 drives the transformation of benign adenocarcinomas into aggressive, metastatic NSCLC. Inhibition of FOXM1 has been shown to reduce the invasion of mucinous adenocarcinoma lung cancer cells. Furthermore, the FOXM1 transcription factor has a binding site within the promoter of an oncogene, anterior gradient protein two homolog (AGR2), which is associated with p53 tumor suppressor protein, inducing its expression [84]. By upregulating SNAIL, an EMT phenotype promoter, FOXM1 drives tumor invasion and metastasis [85]. FOXM1 expression has been shown to increase resistance to radiotherapy in lung cancer cells (A549 and H1299) by inducing kinesin family member 20 (KIF20A), a member of the Hedgehog (Hh) cascade signaling associated with tumor progression and metastasis. FOXM1 activates the KIF20A promoter and regulates its expression at the transcriptional level. Inhibition of FOXM1 downregulates KIF20A, increases radiotherapy resistance, and inhibits radiation-induced EMT [86]. Later studies demonstrated that FOXM1 overexpression leads to resistance to gefitinib, an EGFR small molecule inhibitor used as first-line therapy to treat NSCLC in lung cancer cell lines and primary human lung cancer cells. Furthermore, inhibition of FOXM1 increases the sensitivity of lung adenocarcinoma to gefitinib. FOXM1 is co-overexpressed with MET and protein kinase B (AKT) in lung adenocarcinoma tumors [87]. FOXM1 and MET seem to cooperate in this context: while FOXM1 binds directly to the MET promoter upregulating its expression, MET activates PI3K/AKT signaling through the phosphorylation of AKT, which in turn upregulates FOXM1 expression. Pharmacological inhibition of the MET/AKT axis reduces FOXM1 levels in lung adenocarcinoma cells, suggesting that MET/AKT is a critical target for inhibiting FOXM1 in lung cancer [87]. A study on NSCLC (H1299 and PC9 cells) showed that FOXM1 selectively increases PD-L1 expression by directly binding its promoter. Selective knockdown of FOXM1 mRNA, using a specific siRNA, reduced PD-L1 expression and proliferation and induced apoptosis in NSCLC cells [53]. A similar observation was reported in vivo, indicating that FOXM1 is a promising target for inhibiting PD-L1 expression and cell proliferation in NSCLC [53].

### 4.5. Pancreatic Cancer

Pancreatic cancer is the seventh leading cause of cancer-related deaths worldwide, with an overall survival of 6 months. As in lung cancer, pancreatic cancer cells are also positively regulated by the feedback loop between FOXM1 and hepatocyte growth factor (HGF)/MET signaling [88]. FOXM1 transcriptionally regulates MET. HGF/MET signaling blocks the transcriptional activity of FOXM1, indicating the existence of a positive feedback loop between FOXM1 and MET. Moreover, a similar effect has been observed in other cancers, such as lung cancer [87]. Thus, targeting the FOXM1–HGF/MET axis is considered an opportunity to develop new anticancer drugs for pancreatic cancer [88].

## 5. In Vivo Therapeutic Targeting the *FOXM1* Gene Using ncRNA in Tumor Models

In recent years, the recognition of FOXM1’s pivotal role in driving tumor progression, metastasis, drug resistance, and growth in solid tumors has sparked considerable interest in developing small molecule inhibitors targeting FOXM1 for potential therapeutic applications in patients with solid cancers [46,58,89,90,91,92,93,94,95]. In animal tumor models, targeting of FOXM1 by genetic or pharmacologic (i.e., small molecule) inhibitors demonstrated that FOXM1 is an important driver of tumor growth and progression in aggressive solid cancers. We validated FOXM1 for the first time as a critical therapeutic target in TNBC using genetic siRNA technology, resulting in significant inhibition of TNBC tumor xenografts in mice [62], suggesting that that FOXM1 represents an Achilles heel for targeting TNBC.

### 5.1. Genetic Targeting of FOXM1

Non-coding RNA (ncRNA)-based therapies, including microRNAs (miRNAs) and small interfering RNAs (siRNAs), represent a promising new class of targeted therapies for human diseases. These small RNA molecules, typically 18–24 nucleotides in length, bind specifically to target mRNAs and suppress their expression, making them highly effective tools for silencing oncogenic gene and protein expression [96,97,98]. Recently, first-in-human miRNA therapeutics were found to be safe in phase I clinical trials and moved to phase II clinical trials for advanced cancers, including TargomiR (miR-16 therapy) for mesothelioma [99], Cobomarsen (anti-miR-155) for leukemia/lymphoma [100], and Miravirsen (anti-miR-122) for individuals with Hepatitis C infection [101]. These results indicate that a miRNA-based approach could be feasible in an effort to control FOXM1 expression in solid cancers [102,103].

Our own research has demonstrated that both siRNA and miRNA-based therapeutics, such as miR-34a, can effectively target FOXM1 and inhibit its expression in in vivo studies in mice [62,104]. In preclinical studies using two orthotopic triple-negative breast cancer (TNBC) xenograft models (MDA-MB-231 and MDA-MB-436), systemic intravenous administration of FOXM1-specific siRNA or miR-34a encapsulated in lipid nanoparticles significantly delayed tumor growth. These treatments inhibited the FOXM1/eEF2K axis, reduced intratumoral proliferation and angiogenesis, and induced apoptosis (Table 1) [104]. Additionally, miR-802 has emerged as a potential tumor suppressor in breast cancer [105]. It directly targets FOXM1, inhibiting cell viability, proliferation, and cell cycle progression in MCF-7 breast cancer cells. In animal models, transfection of MCF-7 cells with miR-802 resulted in reduced tumor growth (Table 1) [105]. Similarly, miR-877-5p has been identified as a tumor inhibitor, with overexpression of miR-877-5p leading to suppression of tumor growth in vivo by targeting FOXM1 at both the mRNA and protein levels (Table 1) [30].

These findings highlight the therapeutic potential of ncRNA-based strategies for targeting FOXM1 in solid cancers, offering a promising approach to controlling tumor growth and progression.

### 5.2. Pharmacological Targeting of FOXM1 Small-Molecule Inhibitors

The development of pharmacological inhibitors for FOXM1 has proven challenging due to the complexity of its structure and function. Several strategies have been explored, including the development of small-molecule inhibitors and peptidomimetics, primarily targeting FOXM1’s DNA-binding domain (DBD) to disrupt its interaction with gene promoters [93,106]. Another approach focuses on inhibiting upstream regulators, such as kinases and coactivators, which contribute to FOXM1 activity [42,106,107]. Various compounds have been identified as potential FOXM1 inhibitors, including natural agents like thiostrepton, honokiol, and siomycin A, as well as direct inhibitors such as SR-T100, FDI-6 (Forkhead Domain Inhibitory Compound 6), RCM-1 (Robert Costa Memorial drug-1), DFS, and XST-119 (Table 2) [107]. While these compounds have shown promise, they often suffer from limitations in specificity and potency, with some exhibiting off-target effects that impede their advancement into clinical trials. For instance, thiostrepton, although a potent FOXM1 inhibitor, also displays non-specific activity, including inhibition of proteasomal function and disruption of mitochondrial protein synthesis [106,107].

Thiostrepton inhibits the transcriptional activity of FOXM1 and its expression through the inhibition of proteasomal activity in cancer cells. Because known proteasomal inhibitors have also been shown to reduce FOXM1 expression, data suggest that thiostrepton not only inhibits FOXM1 expression but also suppresses its transcriptional activity. In NSCLC, thiostrepton has been shown to reduce PD-L1 expression through the downregulation of FOXM1 and inhibit the proliferation of tumor cells. This study showed that FOXM1 selectively upregulates PD-L1 expression by directly binding to the PD-L1 promoter. It was also demonstrated that thiostrepton regulates the cell cycle in H1299 and PC9 cells by increasing the sub-G0/G1 population and decreasing the S-phase population. In mouse models with H1299 and PC9 tumor cells, thiostrepton treatment led to decreased tumor size and inhibition of FOXM1 and PD-L1 expression in tumor xenografts [53].

Honokiol is a small molecule inhibitor that binds to the transactivation domain of FOXM1 and inhibits FOXM transcriptional activity by preventing its binding to DNA, leading to the suppression of cell proliferation of breast, lung, pancreatic, and prostate cancers. In addition to its anti-proliferative activity, it exerts a synergistic effect with chemotherapeutic agents through the inhibition of NF-kB activity [112]. In prostate cancer and TNBC, honokiol inhibits FOXM1 but does not cause proteasomal degradation. Halasi et al. indicated that chemical analogs of honokiol did not inhibit FOXM1 [112].

Siomycin A acts as a thiazole antibiotic that inhibits FOXM1’s proteasomal activity and transcriptional activity. In cancer cells, it reduces the expression of anti-apoptotic proteins like Bcl-2, Mcl-1, and caspase-3 cleavage, leading to increased apoptosis in A549 cells [120]. In cholangiocarcinoma cells, siomycin enhanced the activity of 5-fluorouracil (5-FU) chemotherapy.

**Figure 4:**
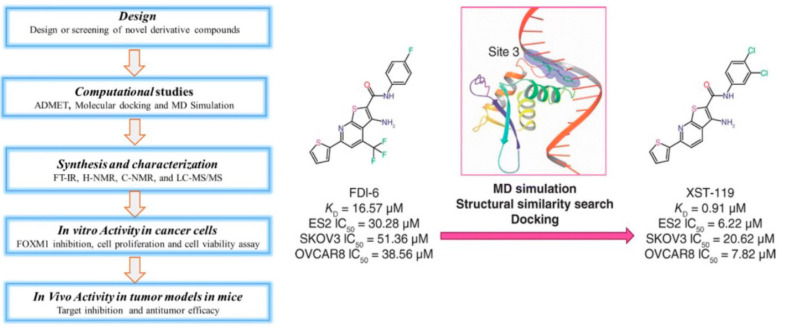
Rational and structure-based drug design. Structure-based virtual screening leads to the design of novel and more potent FOXM1 inhibitors [117]. Based on the results of molecular docking studies, known FOXM1 inhibitors such as FDI-6 could be the basis for computer-aided drug design and virtual screening and identification of novel molecules (i.e., XST-119). A novel FOXM1 inhibitor, XST-119, has a significantly higher affinity for FOXM1 and antiproliferative activity than FDI-6 [117].

FDI-6 (Forkhead Domain Inhibitor-6) directly binds FOXM1 (Figure 4) and inhibits its DNA-binding ability, thereby suppressing cell proliferation (IC50: 1–11 μM), spheroid formation, migration, and invasion and inducing apoptosis in TNBC cells. Additionally, FDI-6 reduces the expression of cyclin B1, Snail, and Slug, which are associated with FOXM1-induced EMT. The combination of FDI-6 and doxorubicin or the PARP inhibitor Olaparib increases cell cytotoxicity and apoptosis, suggesting that this combination with FDI-6 may increase the effectiveness of chemotherapy and PARP inhibitors. In vivo studies showed that FDI-6 (60 mg/kg, IP injection) suppresses the growth of TNBC tumor xenografts in mice models [114]. FDI-6 reduces cell survival by inducing the expressions of N-Ras and phosphoprotein kinase Cδ (p-PKCδ) (S664) in ovarian cancer (HeyA8) cells. The addition of tipifarnib or rottlerin (nonspecific PKCδ inhibitors) to FDI-6 was more effective in reducing the growth of HeyA8 cells compared to FDI-6 alone [121].

RCM-1 induces FOXM1 proteasomal degradation by increasing its ubiquitination, leading to its degradation, and blocks its nuclear localization in various epithelial cells and in a mouse model of goblet cell metaplasia. RCM-1 suppresses the clonogenicity of cancer cells in vitro, delays tumor cell proliferation, and increases apoptosis in vitro [116]. In an in vivo setting in mice, RCM-1 suppresses tumor growth by inhibiting FOXM1 [116]. RCM-1-mediated inhibition of FOXM1 expression leads to cell cycle arrest and suppression of cell cycle progression.

DFS (Lignan (-)-(2R,3R)-1,4-O-diferuloylsecoisolariciresinol), is a natural compound isolated from the plant *Alnus japonica* (*Betulaceae*). DFS has been shown to suppress FOXM1 mRNA expression and FOXM1-driven genes [118]. The FOXM1/β-catenin axis mediates stemness properties and tumorigenesis of glioma stem cells [118]. DFS reduces the expression of β-catenin-mediated genes through suppression of FOXM1 expression in colon cancer [122].

XST-119 has been shown to interact with the FOXM1 surface by plasmon resonance (Figure 3). In addition, recent studies revealed that XST-119 has strong in vitro antiproliferative and in vivo antitumor activities in a tumor xenograft mouse model [117]. The affinity and antiproliferative activity of XST-119 on FOXM1 is significantly higher than FDI-6.

KC12, a new benzothiazole derivative, was synthesized by us [119] and structurally confirmed through FT-IR, H-NMR, C-NMR, and LC-MS/MS analyses. In in silico studies incorporating ADMET analysis, compound KC12, with pharmacokinetic and drug-likeness properties, was identified. In silico molecular docking and MD simulation studies further highlighted the strong interaction of KC12 with critical amino acids (N283 and R286), inhibiting FOXM1-DBD. Evaluations of cytotoxic activity and colony formation in the TNBC cell line (MDA-MB-231) at approximately 10 μM were very promising for KC12 [119].

With regard to designing FOXM1 inhibitors, it is critical to target their DNA binding ability, which may provide better therapeutic efficacy. It is well established that three conserved amino acid residues, including N283, R286, and H287, play crucial roles in the interaction between the FOXM1 protein and DNA [123,124,125,126]. H287 forms direct and indirect hydrogen bonds with the DNA bases [123,126]. Perturbation of this hydrogen-bonding network, through H287 mutation, reduces the binding affinity of FOXM1 to DNA. Furthermore, N283 exhibits two conformations: vertical, engaging in two hydrogen bonds with DNA, and horizontal, forming minimal DNA interactions but engaged in two hydrogen bonds with R286. Mutations in N283 decrease FOXM1’s DNA binding affinity, while mutations in R286 abolish its DNA hydrogen-bond formation, potentially favoring the vertical conformation of N283 and enhancing its DNA interactions [123,124,125,126]. Understanding the structural and functional significance of these amino acid residues is critical when designing the interaction of FOXM1 inhibitors with the DNA-binding domain and selecting highly effective inhibitors.

Targeted protein degradation (TPD) is an emerging approach for therapeutic targeting of proteins that are challenging to target with conventional small molecules. An important class of molecules that can modulate such proteins via TPD are proteolysis-targeted chimera (PROTAC) protein degraders. These are heterobifunctional small molecules consisting of two ligands joined by a linker: one ligand recruits and binds a protein of interest (POI), while the other recruits and binds an E3 ubiquitin ligase [127]. A peptide-based PROTAC was developed to facilitate the recruitment of E3 ubiquitin to FOXM1 for the purpose of degrading FOXM1. Moreover, FOXM1-PROTAC inhibits the proliferation, migration, and invasion of cancer cells, including in in vitro HepG2 and MDA-MB-231 models. FOXM1-PROTAC suppresses tumor growth in in vivo HepG2 and MDA-MB-231 cells. The toxicity is quite low compared to normal tissue. On the other hand, the biodistribution of injected FOXM1-PROTACs showed that p-PROTACs showed strong performance in specific targeting with no significant side or “off-target” effects in tissues [128]. PROTAC PU7-1, targeting FOXM1 through the involvement of USP7 (Ubiquitin Specific Peptidase 7), significantly reduces FOXM1 protein levels in breast cancer cells and inhibits cell proliferation in MDA-MB-468 (IC50 1.8 μM) and BT549 (IC50 2.8 μM) TNBC cells. More important, this approach also suppresses tumor growth in in vivo tumor xenograft models in mice [129].

Although FOXM1 is expressed at low levels in adult differentiated tissues, it is expressed in rapidly proliferating normal tissues such as the testis, thymus, and bone marrow. Therefore, FOXM1-targeted therapies may possibly have some side effects in these tissues, and interfere with the functionality of some tissues and the regeneration or healing of injured tissues [10]. Thus, targeting FOXM1 in tumor tissue using approaches such as tumor-targeted nanocarriers may reduce these possible side effects.

## 6. Conclusions and Future Directions

Recent research has underscored the essential role of FOXM1 oncogenic signaling in driving tumorigenesis and the progression of aggressive solid cancers such as TNBC, pancreatic, lung, and brain cancers. FOXM1 activation induces the expression of genes linked to the hallmarks of cancer, cell proliferation, invasion, tumor growth, resistance mechanisms, immune escape, angiogenesis, and disease advancement. In vivo experiments have validated FOXM1 as a molecular target and its inhibition effectively curtails the growth of tumors, including TNBC. Despite the absence of FDA-approved FOXM1 inhibitors, various small molecule inhibitors and ncRNA molecules, such as siRNA and miRNAs (miRNA-34a and miR-802), have demonstrated therapeutic efficacy in animal models. However, current FOXM1 inhibitors often lack specificity and potency and have off-target effects, thus, none have progressed to clinical trials. Future investigations should prioritize the discovery of selective, potent, and safe FOXM1 inhibitors for clinical translation. Moreover, FOXM1-targeted therapies hold the potential to augment the effectiveness of conventional and targeted cancer treatments, including checkpoint inhibitors. Computational chemistry and molecular docking studies with the help of artificial intelligence and machine learning will be the key to the identification of novel, potent, and selective FOXM1 inhibitors for the development of effective and safe clinically applicable strategies. Thus, the development of safe and efficacious FOXM1 inhibitors is expected to provide a significant impact on patient survival and clinical outcomes and enhance the clinical management of FOXM1-driven solid cancers.

## Figures and Tables

**Table 1:** MicroRNAs used in in vivo tumor models against FOXM1.

MicroRNA	Target Tumor	Effects	References
miR34a	TNBC	Inhibits proliferation and angiogenesis, induces apoptosis	[104]
miR-802	Breast cancer	Inhibits cell viability, proliferation, cell cycle	[105]
miR-877-5p	NSCLC	Inhibits tumor growth	[30]

**Table 2:** FOXM1 inhibitors used in in vitro and in vivo tumor models.

Inhibitor	Inhibitor Type	Target Tumor	Effective Doses	References
Thiostrepton	Natural cyclic oligopeptide antibiotic	Breast, Liver cancer, Melanoma	Breast 0.5–1.8 μM Liver 1.8–6 μM Melanoma 1–2.5 μM	[108,109,110,111]
Honokiol	Polyphenol (neolignan biphenols)	Prostate, Pancreatic, Breast cancer	Prostate 60 μM Pancreatic 60 μM Breast 60 μM	[112]
Siomycin A	Thiopeptide antibiotic	Breast, Liver cancer, Melanoma	Breast 0.5–1.8 μM Liver 1.8–6 μM Melanoma 1–2.5 μM	[109,110,111]
SR-T100	*Solanum incanum* extract	Ovarian cancer	Ovarian 2.5–10 μM	[113]
FDI-6	Chemical inhibitor	Breast, Lung cancer	Breast 4 μM Lung 20 μM	[114,115]
RCM-1	Chemical inhibitor	Melanoma, Lung cancer, Breast cancer, Prostate cancer	Melanoma 1–5 μM Lung 1–5 μM Breast 5–10 μM Prostate 1–5 μM	[116]
XST-119	Chemical inhibitor	Ovarian cancer	Ovarian 0.9–10 μM	[117]
DFS	Natural inhibitor	Brain cancer (GBM), TNBC	GBM 5–10 μM TNBC 5–7.5 μM	[118]
KC12	Small mol. inhibitor	TNBC	TNBC 6.13 μM	[119]

## Abbreviations

ADAM17	ADAM Metallopeptidase Domain 17
ADMET	Absorption–Distribution–Metabolism–Excretion–Toxicity
AGR2	Anterior gradient protein two homolog
Akt	Protein kinase B
APC	Anaphase-Promoting Complex
Arf/CDKN2A	Cyclin-Dependent Kinase Inhibitor 2A
ATM	Ataxia telangiectasia
BC	Breast cancer
Bcl-2	B-Cell Lymphoma 2
BRCA2	Breast cancer 2
Cdc25	Cell division cycle 25
CDK	Cyclin-Dependent Kinase
CDH1	Cdc20 homolog 1
CENP	Centromere Protein
CHK2	Checkpoint kinase 2
CISR	Cisplatin-resistant
CREB	cAMP-Response Element Binding Protein
CTCF	CCCTC-Binding Factor
CTLA4	Cytotoxic T-lymphocyte-associated protein 4
DBD	DNA-binding domain
DNA	Deoxyribonucleic acid
DUB	Deubiquitinating enzyme
E2F	Early region 2 binding factor
E2F1	Early region 2 binding factor 1
E3 Ligase	E3 ubiquitin–protein ligase
eEF2K	Eukaryotic elongation factor 2 kinase
EMT	epithelial–mesenchymal transition
ERβ1/EGFR	ErbB-1, also named epidermal growth factor receptor
ERK	extracellular signal-regulated kinase
ER	Estrogen
FDA	Food and Drug Administration
FDI-6	Forkhead Domain Inhibitor-6
FHRE	Forkhead elements
FOX	Forkhead box
FOXM1	Forkhead box M1
FT-IR	Fourier transform infrared spectroscopy
GATA3	GATA Binding Protein 3
GBM	Glioblastoma multiforme
GLI1	Glioma-Associated Oncogene Homolog 1
H	Histidine
HCC	Hepatocellular carcinoma
HER2	Human epidermal growth factor receptor 2
HGF	Hepatocyte growth factor receptor
HH	Hedgehog
HIF1α	Hypoxia-Inducible Factor 1 alpha
ITGB1	Integrin subunit beta 1
IPO	Importin-7
JNK	Jun N-terminal kinase
KIF20A	Kinesin family member 20
LC3	Microtubule Associated Protein 1 Light Chain 3 Alpha
LC-MS	Liquid chromatography–mass spectrometry
lncRNAs	Long noncoding RNAs
MAPK	Mitogen-activated protein kinase
MCF7	Michigan Cancer Foundation-7
Mcl-1	Myeloid Cell Leukemia 1
MD simulation	Molecular dynamics simulation
MELK	Maternal embryonic leucine zipper kinase
MEK	Mitogen-activated protein kinase
MET	Mesenchymal Epithelial Transition
miR/miRNA	microRNA
MMP	Matrix metalloproteinase
MRE11	Meiotic recombination 1
mRNA	Messenger RNA
ncRNA	Non-coding RNA
N	Asparagine
NF-kB	Nuclear factor kappa B
NMR	Nuclear magnetic resonance spectroscopy
n-Ras	Neuroblastoma rat sarcoma virus
NRD	N-terminal repressor domain
NSCLC	Non-small cell lung cancer
NRFM	Negative regulators of FOXM1
OS	Overall survival
PARP	Poly (ADP-ribose) polymerase
p27/Kip1	Cyclin-Dependent Kinase Inhibitor 1B
PD1	Programmed Cell Death Protein 1
PD-L1	Programmed Cell Death Ligand 1
PVT1	Plasmacytoma variant translocation 1 gene
PLK	Polo-like kinases
POI	Protein of interest
PPI	Protein–protein interaction
p-PKCδ	Phosphoprotein kinase Cδ
PR	Progesterone
PROTAC	Proteolysis-targeted chimera
PTEN	Phosphatase and Tensin Homolog
PTM	Post-translational modification
PVT1	plasmacytoma variant translocation 1 gene
R	Arginine
RAD51	RADiation sensitive protein 51
RAF	Rapidly Accelerated Fibrosarcoma
Rb	Retinoblastoma protein
RCM-1	Robert Costa Memorial drug-1
RFC5	Replication Factor C Subunit 5
S	Serine
siRNA	Small interfering RNA
SLUG	Zinc finger protein SNAI2
SMAD3	Mothers against decapentaplegic homolog 3
Snail	Zinc finger protein SNAI1
Sox2	SRY-Box Transcription Factor 2
SRC	Src proto-oncogene
SRT-100	Solanum incanum extract
STAT3	Signal transducer and activator of transcription 3
SUMO	Small Ubiquitin-like Modifier
T	Threonine
TAD	Transactivation domains
TMZ	Temozolomide
TNBC	Triple-negative breast cancer
TGF-*β*	Transforming Growth Factor Beta 1
TP53	Tumor Protein P53
TPD	Targeted protein degradation
XIAP	X-linked inhibitor of apoptosis protein
XRCC1	X-ray repair cross-complementing protein 1
IBE2C	Ubiquitin-conjugating enzyme E2C
U87MG	Uppsala 87 Malignant Glioma
U373	Uppsala 373
USP7	Ubiquitin Specific Peptidase 7
USP21	Ubiquitin-specific protease
VEGFA	Vascular endothelial growth factor A
VHL	Von Hippel–Lindau syndrome
WDR5	WD repeat domain 5
WNT	Wingless-related integration site
5-FU	5-fluorouracil
5-HT7	5-hydroxytryptamine
